# Palliative Reconstructive Surgery: Contextualizing Palliation in Resource-Poor Settings

**DOI:** 10.1155/2014/275215

**Published:** 2014-10-30

**Authors:** Peter M. Nthumba

**Affiliations:** Plastic, Reconstructive and Hand Surgery Unit, AIC Kijabe Hospital, Kijabe 00220, Kenya

## Abstract

*Introduction*. Palliative care in Kenya and the larger Sub-Saharan Africa is considered a preserve of hospices, where these exist. Surgical training does not arm the surgeon with the skills needed to deal with the care of palliative patients. Resource constraints demand that the surgeon be multidiscipline trained so as to be able to adequately address the needs of a growing population of patients that could benefit from surgical palliation. *Patients and Methods*. The author describes his experience in the management of a series of 31 palliative care patients, aged 8 to 82 years. There were a total of nine known or presumed mortalities in the first year following surgery; 17 patients experienced an improved quality of life for at least 6 months after surgery. Fourteen of these were disease-free at 6 months. *Conclusion*. Palliative reconstructive surgery is indicated in a select number of patients. Although cure is not the primary intent of palliative surgery, the potential benefits of an improved quality of life and the possibility of cure should encourage a more proactive role for the surgeon. The need for palliative care can be expected to increase significantly in Africa, with the estimated fourfold increase of cancer patients over the next 50 years.

## 1. Introduction

The World Health Organization (WHO) defines palliative care as “an approach that improves the quality of life of patients and their families facing the problem associated with life-threatening illness, through the prevention and relief of suffering by means of early identification and impeccable assessment and treatment of pain and other problems, physical, psychosocial and spiritual” [1]. Autonomy, comfort, and dignity are essential elements of palliative care.

Palliative care should therefore be administered in a respectful, open, and sensitive manner, with the understanding that this care is patient-focused and is driven by patient and family needs. Open and sensitive communication constitutes the backbone of successful surgical palliative care, benefiting the patient, relatives, and physician [2, 3].

The definition/attributes of the target population have not been agreed on, but the following attributes have been proposed: illnesses that are progressive, unresponsive to treatment, or advanced and life-threatening. There is also no agreement as to when palliative care for a given patient should be instituted. A number of authors believe that palliative care should be initiated when curative treatment is no longer feasible, while others consider that such care should be introduced early in the course of any life-limiting illness [2–4]. Palliative care must be tailor-made for the individual patient, understanding and optimizing the dynamic interactions within the “palliative triangle” of patient, family, and physician as essential to improve patient outcomes [5].

Education of surgeons in palliative care is only now gradually finding its way into training programs in high income countries (HICs) but remains a far cry in low and middle income countries (LMICs) [6]. Palliative care domains in which programs have been successfully implemented within surgical curricula include pain management, nonpain symptom management and communication skills. Medicolegal, psychosocial, cultural, and spiritual aspects of care, as well as hospice care and referrals, are other aspects of teaching that surgical trainees in HICs have been exposed to. The need for surgical competencies in palliative care nevertheless remains a significant challenge in any setting [6, 7].

## 2. Definitions

Hui et al. found a lack of definitional clarity for many important terms in the supportive and palliative oncology literature, highlighting the need for standardizing terminology and definitions [8].

### 2.1. Palliative Care

Palliative care, an evolving specialty, includes the goals of enhancing the quality of life for patient and family, optimizing function, helping with decision making, and providing opportunities for personal growth, and not merely “not cure-oriented” care, as originally envisaged. Its breadth includes symptom control, psychosocial and spiritual care, communication, decision making, and end-of-life care [2, 8].

Palliative care, irrespective of specialty, is unified by the single goal of providing dignity to the patient: hope, respect, and value. It is care, not for the dying, rather care provided to people with serious and even life-threatening conditions, to which some will succumb [9].

### 2.2. Palliative Surgery

A widely accepted definition is central to the evolution of the discipline, as this would enable the development of standards, audits, research, and outcome measures, which in turn lead to improved patient care. There is currently no consensus on the definition of palliative surgery: authors differ suggesting definitions based on preoperative criteria, postoperative criteria, and even patient prognosis [4]. While palliative surgery may result in cure, the intent is the transformation of the life of the individual by providing an intervention that offers the best quality of life for the remainder of that individual's life [6].

### 2.3. Outcomes

Outcomes relate to the ability of patients to perform tasks that are meaningful, practical, and sustainable over time, outside the clinical environment.

Proper patient selection is perhaps the single-most important variable when offering palliative surgical interventions. Poor performance status, poor nutrition, and no previous cancer treatment are variables associated with poor patient outcomes following surgery; serum albumin levels and weight loss have been reported to be markers predictive of outcomes [3, 4].

The universal assumption that patients undergoing palliative surgical procedures are at a danger of increased morbidity and mortality when compared to other surgical patients has been challenged by some workers: a study by McCahill et al. showed equivalent morbidity and mortality rates between the two groups of patients [10]. Morbidity and mortality rates for palliative patients undergoing surgery have been reported at between 0% to 21% and 15% to 40%, respectively [4]. Kucukmetin et al. in a meta-analysis of palliative surgery versus medical management of bowel obstruction in ovarian cancer reported a small but statistically significant advantage of palliative surgery [11].

A number of authors have reported excellent outcomes following surgical palliation, even in the presence of metastases, without significant cost, in well selected patients [12, 13]. Other reports have indicated patient benefit from wide resection of tumor, with immediate reconstruction, even in the face of positive margins, and/or potential lack of access to adjunct oncologic treatment [14, 15].

### 2.4. Resectability

A tumor may be considered resectable if a number of conditions are fulfilled: the resection must not destroy any vital structures or function(s) or endanger the life of the patient; it must be feasible to reconstruct the defect so created.

### 2.5. Operability

While a tumor may be resectable, poor patient physical, physiological, or psychological status or the presence of extensive metastases may render surgical intervention impossible or inadvisable, as surgery would not meet any of the desired goals. The principle “*primum non nocere*,” must underlie every planned surgical intervention.

### 2.6. Symptom Relief

Palliative surgery has been used by some authors to denote surgical interventions which in themselves have no bearing to the life of the patient but offer the last possible intervention for an improved outcome, with a number reporting durable symptom relief and improved quality of life following surgical palliation [16, 17]. One proposed definition of surgical palliation emphasizes the relief of present or anticipated symptoms, even if the interventions do not prolong the patient's life [6]. Understandably, surgical palliation for symptom relief has largely been limited to the relief of obstructed viscera, usually using minimally invasive procedures [4].

There are not many reports in literature on palliative surgical procedures and even fewer written from the perspective of a resource-constrained environment; most are case reports [15, 18–22]. Even workers from HICs well appreciate the difficulties in the care of the palliative surgical patient [23]. In an area of surgical practice where protocols are difficult to develop and where clinical trials are absent and may even be considered unethical, developing a protocol, albeit a flexible one, is useful. In a patient-focused management process, the care given must be tailor-made for each individual patient, contextualized within that patient's desires and needs. The author reviewed the reconstructive palliative surgical experience in a rural sub-Sahara African hospital over a period of 48 months (August 2009 to July 2013) and presents the data.

## 3. Patients and Methods

A retrospective chart review of all patients undergoing palliative reconstructive surgical procedures over a 48-month period at the author's institution was performed. The inclusion criteria for patient selection were tumor resectability, patient operability, premorbid lifestyle (the patient must have demonstrated independence in self-care and the basic daily activities of life), and postoperative goal(s) (the patient demonstrates a desire to return to an active/productive life, as may be permitted by the outcome of surgery).

The patients and relatives were also counseled on the possible need for adjuvant therapy (radiotherapy, chemotherapy, or a combined treatment), but ability or willingness to access such treatment did not affect the decision to operate or not to.

Patients who had lung or other distant metastases or who did not fulfill the above criteria did not undergo surgical intervention and were therefore excluded from the study.

## 4. Results

**Figure 1:**
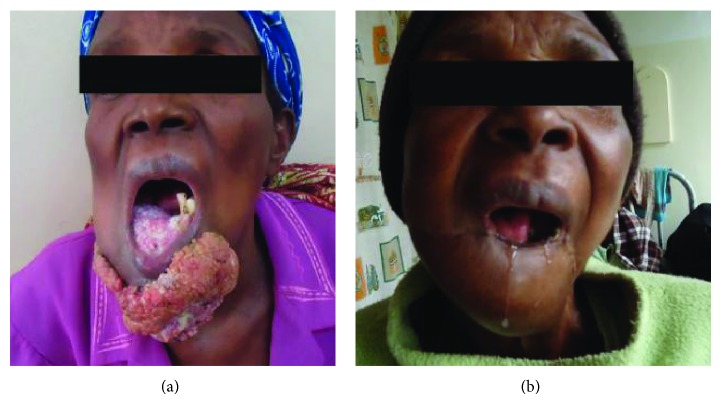
(a) Squamous cell carcinoma of the lower lip in a patient with AIDS on HAART. (b) Post-operative picture.

**Figure 2:**
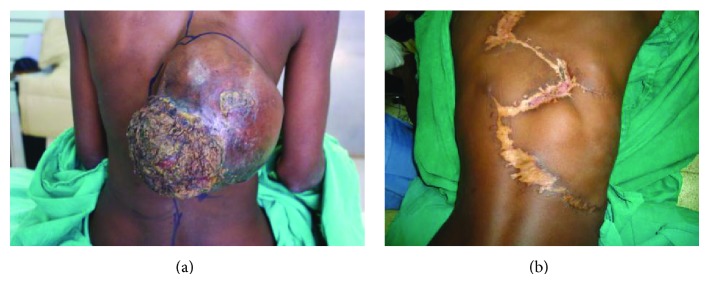
(a) Large posterior trunk soft tissue sarcoma. (b) Recurrence at 2 years.

**Figure 3:**
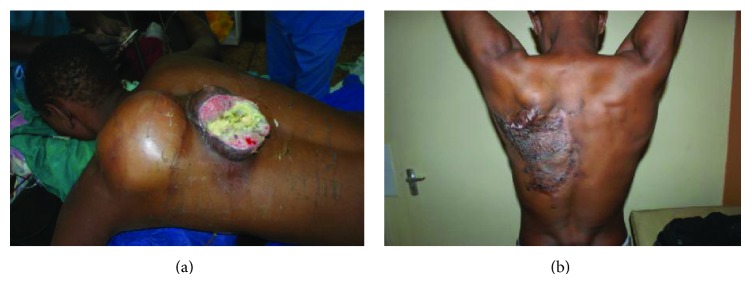
(a) Posterior trunk sarcoma following 3 previous resections. Wide excision, including part of the scapular. Reconstruction using Latissimus dorsi muscle. (b) Same patient a year later. Normal shoulder function and no recurrence. Patient remains asymptomatic at 3 years.

**Figure 4:**
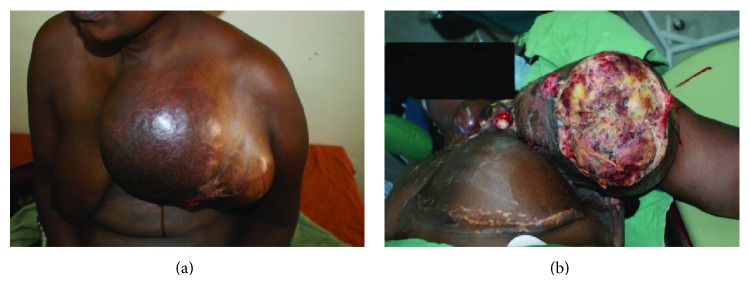
(a) Malignant phyllodes tumor of the breast involving the arm and neck. (b) Recurrence at 9 months. The patient was unable to access medical/radio-oncologic treatment.

**Figure 5:**
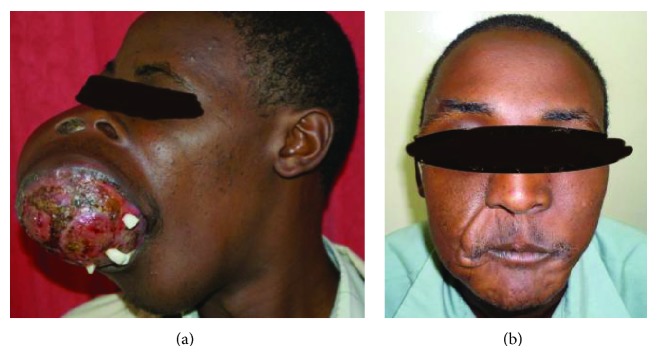
(a) Synchronous osteosarcomas of the maxilla and mandible in a patient placed under palliative medical management. Picture © 2012 Nthumba; licensee BioMed Central Ltd [7]. (b) Postoperative picture at 2 years.

**Figure 6:**
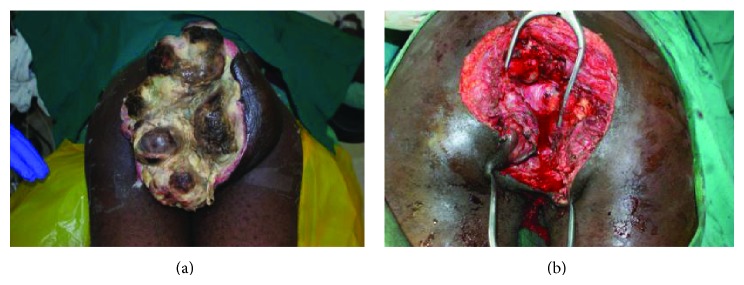
(a) Patient with a perineal sarcoma. (b) Sarcoma excised and a colostomy fashioned. The patient suffered a recurrence at 6 months while awaiting the initiation of medical oncologic therapy.

A total of 31 patients fulfilled the criteria for inclusion into the study. There were 14 females and 17 males, aged 8 to 82 years (Table 1). Representative cases are presented in Figures 1–6.

Of the 31 patients, three had malignant peripheral nerve sheath tumors (2 retroperitoneal and one with extensive bony metastases from an arm lesion). Forty-two percent (11) of the patients had undergone one or more surgical procedures in other institutions; of these, 4 had had two or more attempted resections (Figures 2, 3, and 4).

Postoperatively, three patients died while in hospital, while three others died at home within the first 12 months following surgery; these three had recurrences at the time of death (Figures 4 and 6). Four other patients are suspected to have died—all got lost to follow-up after their first postoperative visit. The status of five patients remains unknown; these were either never seen after discharge from hospital or failed to turn up after being informed of the need for additional surgery because of local recurrence; three of these had come from neighboring countries. Seventeen (55%) had an appreciably improved quality of life for at least 6 months; of these, 14 were disease-free. A total of fourteen patients (48%) were referred for medical oncological treatment; six could not afford the treatment. Only eight patients were treated with chemotherapy, radiotherapy, or a combination. At between 2 and 5 years, 7 patients continue to enjoy a disease-free life, with no recurrence; only two of these received adjuvant chemoradiotherapy. One patient who received chemoradiotherapy for a truncal fibrosarcoma experienced recurrent at 18 months (Figure 2).

One patient developed a recurrence after waiting for 6 months before initiation of chemotherapy for a high grade sarcoma. He had enjoyed a 5-month symptom-free period but experienced system delays before he was able to access chemotherapy, even after timely referral from the author's institution (Figure 6). Two patients with surgically amenable recurrences refused surgery; their current status is unknown.

Four patients had known comorbidities: HIV/AIDS in three (Figure 1) and pathological fractures of a femur and humerus in another. Cor pulmonale was suspected in a fifth.

### 4.1. Bothersome Symptoms

The two or three most bothersome symptoms for which the patient sought surgical intervention were recorded and evaluated. Twenty-two (71%) of the 31 patients identified pain as the symptom that bothered them the most, while foul smell emanating from malignant ulcers was the second most bothersome symptom (58%). Smell was a major hindrance to social interaction. Instructively, four patients desired improved cosmetic appearances to enable them to gain acceptance by their families/communities. One of these had been abandoned by relatives and placed under hospice care but was promptly restored into the family after surgery.

Oral incontinence, odynophagia, and difficulty in feeding were other secondary or tertiary complaints.

Eight patients (26%) experienced poor or fair quality of life postoperatively. These patients could not independently perform some or all basic activities of daily living.

## 5. Discussion

Humans are social beings and the search for well-being often involves the individual whose health or life is at risk, the immediate family, and the community. There is often a complex interplay of variables that influence the health-seeking behavior of people: the individual, sex and age, faith and belief systems, their education, and that of their parent(s) and siblings, and/or spouse, and finally the culture and community from which they come. The available resources and access to quality healthcare services, amongst many other factors, interact to impact the outcome of illnesses/diseases afflicting patients. All these factors have a major bearing on the evolution of terminal illnesses and their management in patients within any society, but perhaps more so in sub-Saharan Africa.

The surgeon in rural sub-Saharan Africa is therefore not infrequently confronted by patients with lesions that are at first glance unresectable. The size of the tumor and its presentation, often ulcerated, infected, and exuding a foul smelling discharge, can be overwhelming (Figures 1, 2, 3(a), 4, 5(a), and 6(a)). The reflex healthcare provider response in this environment is predictable: “nothing can be done”; besides the risk of death, the cost of care may be prohibitive, while the projected postoperative quality of life may at best be poor.

Resectability of most lesions with subsequent reconstruction of the resultant defects in the 21st century is possible, especially with the availability of microsurgical skills and equipment. In sub-Saharan Africa, however, such services remain the preserve of the well-to-do in big cities, where access to healthcare services is good. In most of the rest of the region, late presentation as noted above will often lead to patients being labeled, “inoperable,” primarily, in the current author's experience and opinion, because of envisaged difficulties with the reconstruction of postresection defects [7].

Palliative reconstructive surgery must encompass more than just the ability to reconstruct a defect; the surgeon must factor into the treatment the ability or inability of the patient to access other adjuvant therapies that may be required, especially chemotherapy and/or radiotherapy. Further, the availability and accessibility of such supporting services as imaging and histopathology form an important part of the decision making process. Indeed in rural sub-Saharan Africa, these two services should be considered the minimum necessary for one to successfully perform palliative reconstructive surgery: the former defines resectability and the reconstructive requirement of the postexcision defect, while the latter determines the extent of the margins, the completeness of surgical resection, and the likely need for further interventions/therapy.

### 5.1. Quality of Life

The measurement of health-related quality of life (HRQoL) provides the opportunity for an outcome measure in palliative surgical care and may be used in clinical trials of palliative treatment modalities. HRQoL is however a complex concept, defined as an individual's or group's perceived physical and mental health over time. A number of tools are in the process of development, in an attempt to capture this concept as data, allowing for the evolution of clinical scores that would guide palliative surgical care, in achieving optimum HRQoL.

Palliative reconstructive surgery should ideally improve a patient's quality of life, adding value, worth, and improved health to the patient's life, by removing hindrances to these goals. The shortest and safest route to achieving these goals should be taken: the last days of one's life should not be spent in a hospital bed recuperating from surgery, nor should these last days expend all of an individual's lifesavings, turning them and their dependants into paupers. Rather, these should be spent in as much enjoyment as is possible, within the confines of one's home, with loved ones.

Most authors recognize obstruction, hemorrhage, and perforation as the primary indications for surgical palliation. Established palliative surgical procedures include stenting (hepatobiliary system, the esophagus and ureters, etc.) and pinning of pathological fractures (treatment, for pain or preventive), amongst others. The place for palliative reconstructive surgery is not as well defined.

Because of the lack of standardization of the evaluation of clinical outcomes for palliative surgical interventions, different endpoints are currently used as a measure of success of surgical palliation: cost of care, quality of life, need for repeat interventions, surgical morbidity, and mortality and survival [6]. Approximately how long should this “improved quality of life” be to warrant surgical intervention on a palliative surgical patient—a week? A month? A year? This answer is impossible to quantify scientifically, and the response will be different for every patient. Some patients are grateful to be rid of chronic pain or a foul smelling ulcerated mass for an hour of their lives—and for them to live beyond a month and be able to attend to some favorite activity even with the knowledge that death is certain to come at any time, no price can be placed on such a comfort, even in the most resource-constrained environments.

In order to give an objective assessment of outcomes, Hanna et al. proposed a 6-week and 3-month postoperative assessment of quality of life and symptom response to palliative surgical procedures [6].

Symptom control has been the focus of most palliative care experts; symptom resolution following surgery is between 50% and 80%, emphasizing the fact that appropriate patient selection will yield optimal outcomes [24–26]. Indeed Miner suggested that symptom control should overshadow any attempts at improved survival [3]. Other authors have suggested that palliative care patients should live for at least 60 to 90 days for the benefits of surgical intervention to become apparent [4]. All but three of the 31 patients in this series were alive at 90 days (90% survival); of those who survived, only one had little or no improvement in the quality of life. Pain and foul smell were the two most bothersome complaints (71% and 58%, resp.). While surgery resolved the smell, postoperatively, a few patients still experienced some pain.

Seventy-four percent of patients experienced good to excellent quality of life outcomes—these patients were able to perform all their activities of daily living. Their mobility was enhanced, as was their social reintegration.

The Edmonton Symptom Assessment System was developed as a tool to assess the nine most common symptoms in patients undergoing palliative care. The symptoms include assessed pain, tiredness, nausea, depression, anxiety, drowsiness, appetite, well-being, and shortness of breath [27]. It has been modified by some authors to include a tenth patient-specific symptom and has the potential to be modified to suit a given patient population [28]. Although desirable, there is unfortunately no currently available tool that can be used to objectively evaluate outcomes after palliative surgical procedures because of the complexity and diversity of these procedures as well as their intentioned outcomes.

While surgeons may be able to accurately predict life expectancy of patients undergoing palliative surgical procedures, they have been shown to grossly underestimate the success of the procedures, suggesting that a large number of patients who may otherwise benefit from surgical intervention are deprived of such care [4].

An understanding of the term “palliation” becomes important when one grasps the potential implications of such a label for the patient. This label may effectively close access to any further healthcare for the individual, especially in environments/communities where “palliative care” is largely understood to imply “the doctor has nothing more to offer.” The family/community may abandon the individual perhaps from frustration and helplessness, as was the case of one patient in the current series [7].

In the absence of guidelines, the opinion of this author is that practice should be largely defined by experience (skill), access to radiodiagnostic equipment, and the support of a histopathology laboratory. Inability to access finances for the service(s) is a common hurdle, and many patients may be unable to afford chemotherapy or radiotherapy that may be required postoperatively.

### 5.2. Primary Aim of Surgery

Many authors fault palliative interventions whose primary aim is to prolong survival [3, 5]. Authors agree that the quality of life is a more valuable goal than quantity. There is however no contention that a number of interventions designed purely as palliative procedures may result in potential cure or prolonged survival: the classic example is Halstead's radical mastectomy, designed as a palliative procedure for the relief of the pain of breast cancer; instead it became a curative procedure and remained the standard of care for a century.

Based on institutional experience, palliative reconstructive surgery can, with careful selection, be life transforming, giving new meaning to life for this vulnerable group of patients [7, 22]. Although the intent of surgical interventions in the patient population presented here was not cure, 7 (23%) experienced complete resolution of their disease and remain alive with no evidence of recurrence or metastases at between 2 and 5 years postoperatively. These excellent results suggest that, for a carefully selected group of palliative patients, irrespective of stage at presentation or previous clinical decisions, surgical intervention can lead to improved patient quality of life and even potential cure, with a return to premorbid lifestyle.

Past and ongoing hindrances to the development and acceptance of palliative surgery include an untreatable/incurable tumor/condition, concern about prolonged hospitalization, cost, morbidity, and mortality. There is also the argument that offering palliative surgery to an individual may provide a stimulus for misguided hope and desire for prolonged survival and even cure. While these concerns remain real, rather than hindering the development of palliative surgery, they should offer opportunities for research and development, with the ultimate goal of providing the best care for the patient.

Literature is replete with successful reconstruction of postoncologic defect reconstruction; most of these articles, however, detail reconstruction of defects created with the intent for cure, rather than pure palliation [29–31]. Palliative reconstructive surgery, although occasionally heroic in its attempts and extent, must not be focused on the surgeon, community, or family: the patient must remain the focus at all times. The surgeon must remain sensitive and empathetic, offering practical solutions to difficult problems. For the treatment to be a success, the patient and relatives must be party to all decisions made and must own the entire process; they all must understand the diagnosis, treatment, and prognosis, and the surgeon must always steer out of the emotional attitude, maintaining a professionally informed and yet empathetic attitude. Because of the absence of standards of care, clinical decisions will continue to be dependent on the knowledge base and skills of the surgeon.

Although most palliative care teams are multidisciplinary, surgeons are not usually members of the team, as surgery is not considered a credible palliative care option. Patient care has to be in the context of the cultural, spiritual, and social domains of the patient, fields that are difficult to understand, and may even be prohibitive to the average surgeon.

### 5.3. The Palliative Patient in Sub-Saharan Africa

Based on the proposed definitions of palliative care and the time at which it should be initiated, a number of issues arise that beg consideration of the economic status of the region from which such a definition is given. In the experience of the current author, a substantial number of patients that came under his care had been placed under palliative care and were therefore dealing with end-of-life issues. Patients 1 and 5 for example, with a diagnosis of fibrosarcoma and jaw osteosarcoma, respectively, had had palliative care programs initiated for them; surgical intervention had been ruled out as an option. After surgery, however, these two patients returned to their preillness lifestyles within three months. While their disease-free periods cannot be predicted, they are alive with no recurrences at 5 years and 4 years, respectively, and they both have been unable to see an oncologist primarily because they cannot afford any further treatment. The author's institution while having a vibrant palliative care service does not have medical oncology or radiation therapy services and therefore refers all its patients to the national referral hospital in the city of Nairobi, as do most hospitals in Kenya. This referral system leads to an overburdening of needs at the point of service delivery and, understandably, significant delays in the initiation of needed oncologic treatment.

Chemotherapy and radiotherapy are treatment options more consistent with palliation in HICs. This is understandable as most patients presenting with life-threatening illnesses in HICs do so early; in contradistinction in LMICs, late presentation is almost the norm [7, 20–22, 32].

On initial presentation therefore, the surgeon must maintain an empathetic disposition and be willing to consider all options in treating the patient. A patronizing attitude must be avoided as must any suggestions that lay the blame for the poor patient state on the patient or relatives.

Palliative debulking may have to be considered, as no other real options may exist that would allow the patient to experience some autonomy, comfort, and dignity, before their death.

A notable recent development has been the move to provide palliative care in intensive care settings, a paradigm shift in the thinking of care givers. Studies have shown increased survival of some patients who had undergone palliative procedures for symptom control, translating into improved quality of life with prolonged survival [9].

## 6. Conclusion

Palliative reconstructive surgery is indicated in a select number of patients. Although cure is not the primary intent of palliative surgery, the potential benefits of an improved quality of life and the possibility of cure should encourage a more proactive role for the surgeon in palliative care. Additionally, palliative care should find its way into all surgical curricula, so as to empower the surgeon with the necessary skills for this growing need. The need for palliative care can be expected to increase significantly in Africa, with the estimated fourfold increase of cancer patients over the next 50 years.

## Figures and Tables

**Table 1:** Demographics of patients undergoing palliative reconstructive surgery.

	Sex	Age	Main complaints	Comorbidity	Anatomical site	Histological diagnosis	Previous intervention(s)	LOS	Outcome^*^	Quality of life^*^	Medical oncology referrals and post-op status	Recurrence	^**^Status at 6 months
1	M	20	Pain, smell	None	Back	Fibrosarcoma	3 resections	3 weeks	Good	Excellent	Refused referral	*✗*	Alive

2	F	50	Pain, smell	None	Breast, left arm	Malignant phyllodes	2 resections/radiotherapy	2 months	Good	Fair	Refused referral	✓	Alive

3	M	40	Pain, smell	AIDS on HAART	Peno-scroto-perineal	Giant condyloma acuminatum	None	2 months	Good	Excellent	Not referred	✓	Alive

4	M	30	Pain, smell	None	Perineal/pelvic	Poorly differentiated sarcoma	None	3 weeks	Good	Good	Chemotherapy delayed	✓	Alive

5	M	20	Deformity, difficulty eating/drinking	None	Mandible/maxilla	Well differentiated jaw osteosarcomas	Biopsy, palliative care	6 weeks	Excellent	Good	Refused referral	*✗*	Alive

6	F	25	Pain, smell	None	Breast	Poorly differentiated adenocarcinoma	None	1 month	Good	Good	Referred, lost to follow-up	?	Presumed dead

7	M	45	Pain, oral incontinence	None	Cheek	Squamous cell carcinoma of oral cavity	Debridement	2 months	Poor	Poor	Died in hospital awaiting oncology visit	✓	Dead

8	F	19	Pain, unable to ambulate	Pathological fractures femur, humerus	Arms (bilateral)	NF1 with metastatic MPNST	ORIF humerus, femur^+^	1 month	Fair	Poor	Referred, lost to follow-up	?	Presumed dead

9	F	20	Pain, bowel obstruction	None	Retroperitoneum	NF1 with retroperitoneal MPNST	None	1 month	Good	Good	Referred, lost to follow-up	?	Presumed dead

10	M	40	Pain, early satiety	None	Retroperitoneum	NF1 with retroperitoneal MPNST	Plexiform neurofibroma excised	2 weeks	Good	Fair	Referred, lost to follow-up	?	Presumed dead

11	M	40	Pain, deformity	None	Maxilla	Ameloblastic carcinoma	None	3 weeks	Good	Excellent	Chemotherapy	*✗*	Alive

12	M	35	Smell, oral incontinence	None	Mandible/oral cavity	Ameloblastoma with SCC in fistula	None	3 weeks	Excellent	Good	Awaits mandibular reconstruction	*✗*	Alive

13	M	45	Pain, smell	None	Abdominal wall	Fibrosarcoma	Multiple	4 weeks	Excellent	Good	Referred, lost to follow-up	✓	Unknown

14	F	32	Pain, soiling of clothes	None	Neck	Nodal SCC with no primary	Resection and radiotherapy	3 weeks	Excellent	Excellent	Referred, lost to follow-up	✓	Unknown

15	F	22	Pain, odynophagia	None	Neck	High grade glomus jugulare tumor	1 resection	2 weeks	Good	Excellent	Not referred	✓	Alive

16	M	55	Pain, smell	None	Scalp/skull	Sebaceous carcinoma	None	3 weeks	Good	Excellent	Could not afford chemoradiotherapy	*✗*	Alive

17	M	20	Shortness of breath, tiredness	? Cor pulmonale	Extremities, trunk, intrathoracic	Plexiform neurofibromas	None	2 weeks	Poor	Poor	Perioperative mortality	*✗*	Dead

18	M	8	Pain, smell	Malnutrition	Left eye	Retinoblastoma	None	2 weeks	Good	Fair	Chemotherapy, lost to follow-up	?	Unknown

19	F	40	Smell, pain	None	Shoulder	Fibrosarcoma	None	3 weeks	Excellent	Excellent	Chemotherapy	✓	Alive

20	M	35	Smell, pain, deformity	None	Maxilla	Ameloblastic carcinoma	None	3 weeks	Excellent	Excellent	Chemotherapy	*✗*	Alive

21	F	24	Pain, smell, deformity	None	Maxilla	Ameloblastic carcinoma	Debulking of odontogenic keratocyst^+^	4 weeks	Good	Fair	Could not afford chemoradiotherapy	*✗*	Alive

22	F	16	Pain, inability to walk	Groin nodal metastases	Popliteal fossa	Marjolin's ulcer (SCC)	Incision and drainage, groin “abscess”	3 weeks	Good	Excellent	Chemotherapy	*✗*	Alive

23	M	25	Pain, smell	None	Back	Fibrosarcoma	Attempted resection	3 weeks	Excellent	Excellent	Referred, lost to follow-up	*✗*	Unknown

24	F	35	Pain, smell, oral incontinence	AIDS on HAART	Lower lip	Squamous cell carcinoma	None	3 weeks	Good	Good	Lost to follow-up after discharge	?	Unknown

25	M	40	Pain, inability to walk	None	Right thigh	High grade fibrosarcoma	None	4 weeks	Good	Fair	Perioperative mortality	*✗*	Dead

26	F	20	Smell, soiling of clothes	Debridement and STSG	Right elbow	Marjolin's ulcer (SCC)	None	2 weeks	Fair	Good	Radiotherapy	*✗*	Alive

27	M	82	Smell, pain, difficulty opening mouth	Chronic smoker	Mandible, floor of mouth	Squamous cell carcinoma	None	3 weeks	Good	Good	Under follow up	*✗*	Alive

28	F	56	Smell, pain, difficulty opening mouth	HIV on HAART	Lower lip, floor of mouth	Squamous cell carcinoma	None	6 weeks	Good	Good	Radiotherapy	*✗*	Alive

29	F	25	Pain, smell, difficulty opening mouth	None	Neck, cheek, floor of mouth	Squamous cell carcinoma	None	3 weeks	Excellent	Excellent	Radiotherapy	*✗*	Alive

30	F	22	Pain, smell, difficulty moving around	None	Neck, floor of mouth	Liposarcoma	3 resections	2 weeks	Excellent	Excellent	Referred for chemotherapy	*✗*	Alive

31	M	35	Pain, inability to walk	None	Right thigh	Fibrosarcoma	None	2 weeks	Good	Good	Referred for chemotherapy	*✗*	Alive

SCC: squamous cell carcinoma; AIDS: acquired immune deficiency syndrome; HAART: highly active antiretroviral treatment; MPNST: malignant peripheral nerve sheath tumor; ORIF: open reduction and internal fixation; LOS: length of hospital stay.

All previous surgical interventions were performed in other institutions except two^+^.

^*^Immediate outcome/quality of life. Poor: quality of life unchanged; patient functioning at or below preoperative status. Fair: symptoms improved, but patient still complains of pain, or other disability, or no odor but needs to continue with wound dressing changes after surgery. Good: most symptoms gone, and patient independently performs all activities of life but is unable to carry out demanding/manual tasks. Excellent: patient is back to premorbid activities, with no physical restrictions.

^**^Status of patient at 6 months following surgery.

Recurrence at last review: ✓: clinical evidence of recurrence; *✗*: no evidence of recurrence; ?: status unknown.

## Abbreviations

WHO: World Health Organization
HIC: High income country
LMIC: Low and middle income country
MPNST: Malignant peripheral nerve sheath tumor
CTScan: Computerized tomogram of scan
QoL: Quality of life
AIDS: Acquired immune deficiency syndrome
HRQoL: Health-related quality of life.

## References

[1] World Health Organization (2002). *Definition of Palliative Care*.

[2] Pastrana T., Jünger S., Ostgathe C., Elsner F., Radbruch L. (2008). A matter of definition—key elements identified in a discourse analysis of definitions of palliative care. *Palliative Medicine*.

[3] Miner T. J. (2011). Communication skills in palliative surgery: skill and effort are key. *Surgical Clinics of North America*.

[4] Sallnow L., Feuer D. (2010). The role of surgery in the palliation of malignancy. *Clinical Oncology*.

[5] Thomay A. A., Jaques D. P., Miner T. J. (2009). Surgical palliation: getting back to our roots. *Surgical Clinics of North America*.

[6] Hanna N. N., Bellavance E., Keay T. (2011). Palliative surgical oncology. *Surgical Clinics of North America*.

[7] Nthumba P. M. (2012). Osteosarcoma of the jaws: a review of literature and a case report on synchronous multicentric osteosarcomas. *World Journal of Surgical Oncology*.

[8] Hui D., Mori M., Parsons H. A., Kim S. H., Li Z., Damani S., Bruera E. (2012). The lack of standard definitions in the supportive and palliative oncology literature. *Journal of Pain and Symptom Management*.

[9] Dunn G. P. (2011). Surgical palliative care: recent trends and developments. *Surgical Clinics of North America*.

[10] McCahill L. E., Smith D. D., Borneman T., Juarez G., Cullinane C., Chu D. Z. J., Ferrell B. R., Wagman L. D. (2003). A prospective evaluation of palliative outcomes for surgery of advanced malignancies. *Annals of Surgical Oncology*.

[11] Kucukmetin A., Naik R., Galaal K., Bryant A., Dickinson H. O. (2010). Palliative surgery versus medical management for bowel obstruction in ovarian cancer. *The Cochrane Database of Systematic Reviews*.

[12] Wornom I. L., Smith J. W., Soong S. J., McElvein R., Urist M. M., Balch C. M. (1986). Surgery as palliative treatment for distant metastases of melanoma. *Annals of Surgery*.

[13] Hagood C. O., Mozersky D. J., Fite F. W. (1974). Staged palliative resection of recurrent carcinoma of the neck using an extra anatomic carotid bypass. *Surgery*.

[14] Perez F. J. M. (1985). Palliative oncologic surgery. Immediate reconstruction. *Revista Argentina de Cirugia Plastica*.

[15] Adigun I. A., Ogundipe K. O., Bello J. O. (2009). Management dilemma of a recurrent huge fibrosarcoma in a 25-year-old African: a case report. *Journal of Medical Case Reports*.

[16] Morrogh M., Miner T. J., Park A., Jenckes A., Gonen M., Seidman A., Morrow M., Jaques D. P., King T. A. (2010). A prospective evaluation of the durability of palliative interventions for patients with metastatic breast cancer. *Cancer*.

[17] Morel-Fatio D., Lalardrie J. P. (1964). Palliative surgical treatment of facial paralysis. The palpebral spring. *Plastic & reconstructive surgery*.

[18] Nthumba P., Bird G. (2010). Marjolin's ulcer in a spina bifida patient: a case report. *East and Central African Journal of Surgery*.

[19] Nthumba P. M. (2010). Marjolin's ulcers: theories, prognostic factors and their peculiarities in spina bifida patients. *World Journal of Surgical Oncology*.

[20] Nthumba P., Barasa J., Cavadas P. C., Landin L. (2012). Pedicled fasciocutaneous anterolateral thigh flap for the reconstruction of a large postoncologic abdominal wall resection defect: a case report. *Annals of Plastic Surgery*.

[21] Nthumba P. M., Ngure P., Nyoro P. (2011). Giant condyloma acuminatum of the scrotum in a man with AIDS: a case report. *Journal of Medical Case Reports*.

[22] Nthumba P. M. (2011). Squamous cell carcinoma (Marjolin's ulcer) in an orocutaneous fistula of a large mandibular ameloblastoma: a case report. *Journal of Medical Case Reports*.

[23] Merz T., Klein C., Uebach B., Kern M., Ostgathe C., Bükki J. (2011). Fungating wounds—multidimensional challenge in palliative care. *Breast Care*.

[24] Reinhold R. B., Lokich J. J. (1979). Electrocoagulation: palliative surgery to control metastatic cutaneous malignancy. *Journal of Surgical Oncology*.

[25] Pless M., Weinberg U. (2011). Tumor treating fields: concept, evidence and future. *Expert Opinion on Investigational Drugs*.

[26] Klein C., Lang U., Bükki J., Sittl R., Ostgathe C. (2011). Pain management and symptom-oriented drug therapy in palliative care. *Breast Care*.

[27] Bruera E., Kuehn N., Miller M. J., Selmser P., Macmillan K. (1991). The Edmonton Symptom Assessment System (ESAS): a simple method for the assessment of palliative care patients. *Journal of Palliative Care*.

[28] Watanabe S. M., Nekolaichuk C., Beaumont C., Johnson L., Myers J., Strasser F. (2011). A multicenter study comparing two numerical versions of the Edmonton symptom assessment system in palliative care patients. *Journal of Pain and Symptom Management*.

[29] Nouraei S. A. R., Ismail Y., Gerber C. J., Crawford P. J., McLean N. R., Hodgkinson P. D. (2006). Long-term outcome of skull base surgery with microvascular reconstruction for malignant disease. *Plastic and Reconstructive Surgery*.

[30] Sun G.-W., Lu M.-X., Wu W.-M., Hu Q.-G., Yang X.-D., Wang Z.-Y., Wen J.-M., Tang E.-Y. (2011). Reconstruction of oral soft tissue defects with free anterolateral thigh flap. *Zhonghua Zheng Xing Wai Ke Za Zhi*.

[31] Ramkumar A., Francis N. J., Kumar R. S., Kumar S. D. (2012). Bipaddled submental artery flap. *International Journal of Oral and Maxillofacial Surgery*.

[32] Nthumba P. M. (2012). The supraclavicular artery flap: a versatile flap for neck and orofacial reconstruction. *Journal of Oral and Maxillofacial Surgery*.

